# The gap between appointments is where child health is won or lost

**DOI:** 10.1093/haschl/qxag146

**Published:** 2026-06-09

**Authors:** Md Zillul Karim

**Affiliations:** ToguMogu (Pvt.) Limited, 9 Sonargaon Janapath Road, Dhaka 1230, Bangladesh; and Light of Hope Limited, Rezina Garden, 67/A, Dhanmondi 9/A, Dhaka 1 209, Bangladesh

**Keywords:** parental mental health, child health policy, Medicaid, early childhood, parenting support, health infrastructure, pediatric care, parental isolation, digital health, surgeon general

## Abstract

The 2024 US Surgeon General's advisory found that 33% of US parents report high stress levels and 65% experience loneliness. Despite this, the US health system remains organized around the child rather than the parent, with no systematic mechanism for supporting parental confidence between appointments. This commentary argues that the gap between well-child visits, which can stretch to 8 weeks in the newborn period, is where early childhood health is won or lost. This commentary proposes 3 policy changes: that pediatric practices make systematic tiered referrals to parenting support as standard care; that Medicaid and the Children's Health Insurance Program recognize parent-support programs as eligible health investments through appropriate and distinct funding pathways; and that early childhood health metrics incorporate validated measures of parent confidence alongside child outcomes, beginning with programs that already have federal evaluation requirements. Reframing parent support as health infrastructure, not a supplemental service, is the missing policy lever for improving child health outcomes in the United States.

## Teaser

The US health system tracks children carefully. It barely supports the parents raising them. The gap between appointments is where early childhood health is won or lost. Fixing it requires recognizing parent support as health infrastructure, not a supplemental service.

Key Points

- The US health system systematically fails to support parents between clinical appointments, creating a structural gap that screening alone cannot fill.
- Structured, continuous parenting support, delivered through trusted and accessible channels, demonstrably improves health-seeking behavior and reduces parental isolation.
- Three policy changes—tiered referral to parenting support, clarified Medicaid and CHIP funding pathways, and measurement of parent confidence in existing federal programs—would create accountability for a dimension of child health the system currently ignores.

A new mother texts her pediatrician at 3 Am Her 6-week-old will not stop crying. She cannot tell if it is hunger, colic, or something that should send her to the emergency room. The nurse on call helps her through it. The baby settles. The call ends. What follows is 6 more weeks until the 2-month well-child visit, with no structured support and the same uncertainty waiting at every hour the system is not watching. She is not a patient. And the health system, for all its careful monitoring of her child, has no mechanism designed to reach her. I have spent a decade trying to be what that mother needed at 3 Am The lesson is not that the technology is hard. It is that the health system has not decided she is worth reaching.

In August 2024, the US Surgeon General issued a formal advisory on the mental health and well-being of parents. The findings were striking: 33% of US parents report high levels of stress, compared to 20% of other adults. Nearly half say their stress is completely overwhelming on most days. Approximately 65% experience loneliness, compared to 55% of nonparents.^1^ The relevance to child health is direct: children of stressed parents are 4 times more likely to have poor general health and twice as likely to have behavioral or developmental disorders.^1^ Parental stress and isolation are not separate from child health—they are the caregiving environment in which child health is formed. The advisory called for policy changes, expanded community programs, and cultural shifts. What it could not fully answer was a more fundamental question: how do you reach parents before the stress compromises the caregiving environment, and before they ever walk into a clinical setting? That question sits at the center of a structural gap in US early childhood health, one that no amount of additional screening or clinical referrals will close.

The US health system is organized around the child. Pediatric well visits track developmental milestones. Newborn screenings catch metabolic disorders. Immunization schedules are rigorously monitored. What the system does not address is the parent's capacity to deliver on all of that between appointments. This is not a criticism of clinicians. It is a design reality. Clinical systems were built to diagnose and treat. They were not built to be present at 2 Am when a first-time mother cannot tell if her infant's cry is hunger, pain, or something she should worry about. They were not built to guide a father through the developmental window between 6 and 18 months, when early language exposure has documented, lifelong consequences for literacy and cognitive development.^2^ They were not built to deliver the kind of continuous, contextual, trusted guidance that previous generations received from extended family networks.

The advisory names parental isolation as a central stressor, noting that 77% of single parents experience loneliness.^1^ The multigenerational household, where grandparents and community elders once transmitted parenting knowledge informally, has been replaced by smaller, geographically mobile family units.^3^ The gap between a 2-week newborn visit and a 2-month well-child checkup is 8 weeks. In early developmental terms, it is an eternity.

The policy instinct is to add more touch points: screen for postpartum depression, ask parents how they are doing at well-child visits, and connect them to a referral. The American Academy of Pediatrics and Bright Futures already recommend screening for maternal postpartum depression at well-child visits.^4^ This matters. But it is reactive by design: it identifies distress after it has developed. Parental stress and isolation directly compromise the caregiving environment that shapes a child’s neurological development, making upstream prevention the more consequential policy lever.^1,5^ Yet US early childhood health programs measure outcomes primarily at the child level, as if parental confidence and knowledge are inputs the system can assume rather than conditions it needs to cultivate.

The problem is not a shortage of information. Parents today have more parenting content available than any previous generation. What they lack is trusted, continuous, contextual support: guidance calibrated to their child's specific age and stage, delivered through channels they already use. That is not a clinical function. The health system has not yet found a systematic way to provide it. Trust is built through consistency, not comprehensiveness: parents respond to reliable presence, not information volume. Channel matters more than content: information delivered outside a trusted, familiar channel does not reliably change behavior. Most importantly, the parent is the primary health intervention. Supporting parental confidence and knowledge in the first 5 years is not ancillary to child health: it is the intervention itself. A meta-analytic review of online and digital parenting programs found significant improvements in parenting practices, parental self-efficacy, and child outcomes across diverse populations, with effects sustained beyond the intervention period.^6^ Home visiting programs that build sustained relationships with families, such as the nurse–family partnership, have demonstrated long-term reductions in child maltreatment, improvements in child cognitive development, and gains in maternal economic self-sufficiency across multiple randomized trials.^7^ The evidence is clear. The question is whether US health policy is prepared to recognize structured parenting support as infrastructure rather than a supplement.

Three policy changes would begin to close the gap. Table 1 summarizes the current and proposed state across each domain.

**Table 1.** Current state and proposed policy changes for parenting support in US early childhood health.

Policy domain	Current state	Proposed change
Pediatric standard of care	Parenting support offered reactively, only when problems are detected. No standard referral mechanism at well-child visits	Systematic tiered referrals at well-child visits for all new parents—support intensity proportionate to family need, not uniform enrollment
Medicaid and CHIP coverage	No formal recognition of parent-support programs. Child-directed and parent-directed services not distinguished in policy	Child-directed programs seek EPSDT coverage through CHIP and children's Medicaid. Parent-directed programs seek adult Medicaid behavioral health coverage. Both require CMS guidance and state flexibility
Early childhood health metrics	Program evaluation measures child outcomes only. Parental confidence treated as an assumed input, not a measured condition	Head Start, MIECHV, and Medicaid managed care incorporate a validated parental self-efficacy measure, building on the 2024 CMS Adult Core Set mandate requiring behavioral health reporting

## Recommendation 1: systematic tiered referral to parenting support

Pediatric practices should make universal, systematic referrals to parenting support resources at well-child visits—not just when problems are detected. This is distinct from clinical prescribing: pediatricians already refer families to WIC, home visiting programs, and community health workers as standard care. A warm referral to a parenting support resource at the 2-week newborn visit should be as routine as recommending a car seat. This does not require pediatricians to manage adult clinical care. It also means accepting a real structural limitation: a pediatric referral is a suggestion, not a clinical handoff. The parent must choose to act on it. The silo between pediatric and adult medicine is real and this recommendation does not dissolve it—closing that gap will require referral tracking infrastructure beyond the scope of this commentary.

The resource concern is addressable through a tiered model endorsed by the AAP: most families receive a brief, low-intensity resource while more intensive programs such as home visiting are reserved for families with greater need. This is not a recommendation that every parent enroll in formal parenting classes. A prospective tiered model in Pennsylvania found 78% parent enrollment at low cost using existing community infrastructure.^8^ Critically, this recommendation does not assume CHIP alone bears the cost—as Recommendation 2 addresses, child-directed and parent-directed services follow distinct funding pathways.

## Recommendation 2: clarified funding pathways through Medicaid and CHIP

Funding pathways for parenting support should be clarified across 2 distinct mechanisms. For child-directed services—programs that improve parental knowledge of child development and safe caregiving—coverage through CHIP and children's Medicaid is appropriate and consistent with existing EPSDT authority. CMS guidance explicitly identifies parent and family support services as coverable under EPSDT when necessary to support family-centered care for an eligible child.^9^

For parent-directed services—programs that reduce parental stress and isolation specifically because doing so improves the caregiving environment for children—adult Medicaid behavioral health coverage is the appropriate and distinct pathway. This commentary does not propose replacing clinical mental health treatment. It proposes that structured, evidence-based programs be recognized as a nonclinical complement, justified by child health impact, not by treating the parent as a clinical patient. Conflating these 2 funding streams has historically stalled implementation. Clarifying the distinction allows programs to pursue the right authorization. Both pathways require CMS guidance and state flexibility—the argument is not that this is easy, but that the complexity is worth confronting.

## Recommendation 3: measuring parent confidence in existing federal programs

Early childhood health metrics should incorporate validated measures of parent confidence alongside child outcomes, beginning with programs that already have federal evaluation requirements. Head Start, MIECHV home visiting programs, and Medicaid managed care contracts are natural starting points. The CMS Adult Core Set reporting mandate became mandatory for states in 2024, establishing a precedent for adding parent-level measures to existing accountability frameworks.^10^ Adding one validated instrument—such as the Parenting Sense of Competence scale—to existing evaluation frameworks is a targeted, feasible starting point, not a call for universal mandate.

Each recommendation faces real implementation challenges. Referral infrastructure is uneven across states. Medicaid authorization for both pathways requires CMS guidance and state flexibility. Metric adoption requires buy-in from administrators facing substantial evaluation burdens. The scope-of-practice boundaries between pediatric and adult medicine are real constraints, not bureaucratic obstacles. These challenges are solvable but must be named clearly.

The Surgeon General's advisory signals that parenting is finally being recognized as a public health issue rather than a private responsibility. The policy conversation that follows should resist treating the caregiving environment as a purely clinical problem. Clinical interventions for parents in crisis are necessary. They are not sufficient to reach the majority of parents who are stressed and isolated but not yet in clinical need—and whose children are shaped by that stress every day between appointments.

The building blocks for change are not absent—more than 1400 Federally Qualified Health Centers operate in medically underserved communities, MIECHV funds home visiting in every state, and the CMS Adult Core Set now requires behavioral health reporting. But building blocks are not a system. Connecting them requires CMS guidance, state policy action, Medicaid authorization, and sustained clinical buy-in. None of that is simple. The gap between what we know and what we do is not a technical failure. It is a policy choice. And policy choices can be changed.

## Supplementary Material

qxag146_Supplementary_Data

## Author biography

Md Zillul Karim is co-founder of ToguMogu, a digital parenting and family health platform serving over 200 000 registered families in Bangladesh, and Board Director of Light of Hope Limited. Through ToguMogu and its partner programs, he has reached over 300 000 families across digital health, education, and community-based activities. He has led programs in partnership with UNFPA and Jhpiego, a Johns Hopkins affiliate. ToguMogu was selected as one of 10 global winners of UNFPA's Joint Innovation Challenge 2022, chosen from 300 applications across 61 countries.
